# Multi-Year Registry Study of Elapegademase Treatment in Patients With Adenosine Deaminase Severe Combined Immunodeficiency (ADA-SCID) Requiring Enzyme Replacement Therapy

**DOI:** 10.1007/s10875-025-01873-3

**Published:** 2025-03-27

**Authors:** Morna J. Dorsey, Manish J. Butte, Jay A. Lieberman, Heather Lehman, Tracy Fausnight, Michael D. Keller, Caroline Fradette, Michael S. Hershfield, Tamara C. Pozos, Anna Rozova, Luke A. Wall, Jeffrey J. Bednarski, Teresa K. Tarrant, Hey J. Chong, Bob Geng, Noemi Toiber Temin, Susan S. Laubach, Leo Lin, Talal Mousallem, Jolan E. Walter

**Affiliations:** 1 University of California San Francisco Medical School, San Francisco, CA USA; 2 University of California Los Angeles, Los Angeles, CA USA; 3 University of Tennessee Health Science Center, Memphis, TN USA; 4 University of Buffalo Jacobs School of Medicine and Biomedical Sciences, Buffalo, NY USA; 5 Penn State Health Hershey Medical Center, Hershey, PA USA; 6 Children’s National Hospital, Washington, DC USA; 7 Chiesi Global Rare Diseases, Toronto, ON Canada; 8 Duke University School of Medicine, Durham, NC USA; 9 Children’s Minnesota, Minneapolis, MN USA; 10 Louisiana State University Health Sciences Center and Children’s Hospital New Orleans, New Orleans, LA USA; 11 Washington University School of Medicine, St. Louis, MO USA; 12 University of Pittsburgh School of Medicine, Pittsburgh, PA USA; 13 School of Medicine, University of California San Diego, San Diego, CA USA; 14 University of South Florida, Tampa, FL USA; 15 Johns Hopkins All Children’s Hospital, St Petersburg, FL USA

**Keywords:** Elapegademase, ADA-SCID, Real-world, Safety, Primary Immunodeficiency, Enzyme Replacement

## Abstract

**Purpose:**

The safety and tolerability of elapegademase (elapegademase-lvlr; Revcovi^®^) a PEGylated recombinant adenosine deaminase (ADA), were demonstrated in two Phase 3 clinical trials in the U.S. and Japan in patients with ADA-deficient severe combined immunodeficiency (ADA-SCID). Elapegademase replaced Adagen^®^ (pegademase, a PEGylated bovine ADA) in 2018. This registry study (NCT03878069) was conducted as a post-marketing requirement to bolster the limited safety and effectiveness data on elapegademase in patients with ADA-SCID and to study patients starting on enzyme replacement therapy (ERT) *de novo*.

**Methods:**

Patients were managed by routine clinical care and treating physicians’ judgement from September 2019 to January 2023. Primary endpoints included trough plasma ADA activity and total trough erythrocyte deoxyadenosine nucleotides (dAXP). Secondary outcomes included lymphocyte counts, hospitalizations, infections, and safety outcomes.

**Results:**

Thirty-two patients were grouped as ERT-naïve (*n* = 7; infants and children with no prior ERT [EN]); pegademase-transitioning (*n* = 21; from pegademase to elapegademase [PT]); and patients who had participated in the Phase 3 clinical trial (*n* = 4; STP-2279-002; [STP]). The EN group maintained optimal plasma ADA activity, increased lymphocyte counts, had manageable infections, and had no mortality for up to 30 months while on elapegademase. The STP group and 66.7% of the PT group continued to maintain satisfactory levels of both ADA and dAXP with stable rates of infections and hospitalizations and stable lymphocyte counts for up to 48.6 months. Variability on all measures was seen, but overall, patients did not deteriorate while on elapegademase.

**Conclusion:**

Effectiveness of elapegademase was maintained up to 4 years of use and with no new safety concerns.

**Supplementary Information:**

The online version contains supplementary material available at 10.1007/s10875-025-01873-3.

## Introduction

Adenosine deaminase (ADA), encoded by the *ADA* gene, is a key enzyme in the purine salvage pathway. Inborn genetic variants of *ADA* result in a rare and potentially fatal autosomal recessive disorder, adenosine deaminase-deficient severe combined immunodeficiency (ADA-SCID) [1, 2, 3]. ADA-SCID occurs in approximately 1 in 200,000–1,000,000 newborns [2, 4], accounting for approximately 10–15% of SCID cases [5].

Variants in the *ADA* gene on chromosome 20q12-q13.11 cause a systemic deficiency in ADA expression, with T, B, and NK cells being the most susceptible to accumulation of purine metabolites [1, 2, 3, 6]. The types of mutations are vast, with greater than 70 causative, pathogenic mutations identified in ADA-SCID [1]. The toxic levels of deoxyadenosine nucleotides (dAXP) in these cells results in SCID and a high risk of opportunistic infections [1, 2, 3, 7, 8]. High levels of toxic metabolites can cause non-immunologic manifestations of the pulmonary, skeletal, dermatologic, gastrointestinal, and other systems [6].

The age of onset and severity of the disease are related to the type of variant and its resulting level of ADA expression [9, 10]. ADA deficiency can be further classified as early-onset (i.e. infants and small children) ADA-SCID; or less severe delayed (i.e. usually diagnosed between 1 and 10 years), or late-onset (i.e. diagnosed between 2nd to 4th decades) combined immunodeficiency (ADA-CID) [3]. Typical early onset ADA-SCID presents as a life-threatening disease in the first weeks to months of life with poor growth [3]. In countries where newborn screening is practiced and affected infants are detected early, ADA-SCID may be asymptomatic despite severe lymphopenia [3]. Less severe phenotypes of ADA-CID, which accounts for up to 20% of cases, are rarer than ADA-SCID, and typically present with infections or autoimmunity [3]. Following the inception of newborn screening in the U.S., overall survival of patients with ADA-SCID has nearly doubled [11]. Early diagnosis and prompt treatment of ADA-SCID improves survival, reduces infection risks, and enhances quality of life; if left undiagnosed and untreated, children usually die before 2 years of age [1, 2, 3].

Allogeneic hematopoietic stem cell transplant (HSCT) or autologous hematopoietic stem cell gene addition therapy (HSC-GT) are the only definitive therapies available for patients with ADA-SCID [3, 5]. Enzyme replacement therapy (ERT) is the primary treatment for ADA-SCID (1) until patients can receive HSCT or HSC-GT; (2) when transplant therapy fails to recover immune function to acceptable level [3, 12]; or (3) when definitive therapy is not an option [13, 14]. ERT serves as an exogenous source of functional ADA enzyme [15], which reduces dAXP in lymphocytes and nonimmune cells, promoting immune recovery, reducing severe infections, supporting normal growth, and preventing or reversing organ damage [3, 15, 16]. ERT does not cure the disease, and treatment must be given regularly to maintain detoxification [3].

ERT with Adagen^®^ (pegademase), a PEGylated modified bovine ADA, started in the United States in 1990 and has been used in the EU since the early 2000s. Hundreds of patients worldwide have received pegademase to treat complications of ADA deficiency [12, 17]. Pegademase treatment rapidly increases plasma ADA activity and reduces intracellular dAXP metabolites [18]. More than 80% of ADA-SCID patients who received ADA ERT had increased T, B and/or NK cell counts, generated naïve T and B cells, and improved T cell functions [19].

To reduce the risk of bovine spongiform encephalopathy (BSE) and the dependence on bovine-derived products, elapegademase (Revcovi^®^, elapegademase-lvlr), a PEGylated recombinant bovine ADA replaced pegademase in 2018 [18]. Elapegademase is the only U.S. Food and Drug Administration (FDA)-approved ERT for ADA-SCID in pediatric and adult patients [15]. Compared to pegademase, elapegademase has no risk of transmitting BSE and has a prolonged stability due to its succinimidyl carbonate linker, which does not affect enzyme activity [16, 18, 20].

Due to the ultra-rare nature of ADA-SCID, there is limited clinical experience with elapegademase. In a U.S. Phase 3 clinical trial (STP-2279-002), elapegademase provided therapeutic trough plasma ADA activity, safely maintained metabolic detoxification, and maintained or improved lymphocyte counts in six patients with ADA-SCID who were previously on pegademase therapy (mean elapegademase exposure: 135.7 weeks per patient) [16]. Similarly, in a multicenter, single-arm, open‐label, Phase 3 and post-marketing clinical study of elapegademase in Japan, four patients with ADA-SCID achieved undetectable levels of dAXP, sufficient ADA activity, increased T and B cell numbers, and slightly elevated IgM and IgA immunoglobulin levels [21].

Since the clinical studies with elapegademase were in a small group of patients over a short period of time, additional data collected during patients’ routine clinical care are crucial for further understanding the effectiveness and safety. In a case series of newly diagnosed infants with ADA-SCID, all identified by newborn screening, elapegademase treatment reconstituted B and NK cells in two patients within the first month, followed by naïve T cells one month later [17]. A third patient reconstituted all lymphocyte subsets within the first month of ERT treatment. Erythrocyte dAXP levels declined by Week 17 in all patients. Another study from eight U.S. and five Canadian centers analyzed retrospective data from 17 patients’ medical charts [18]. Most patients with ADA-SCID treated for six months or more with elapegademase increased plasma ADA activity and decreased dAXP. Six ERT-naïve infants treated with elapegademase demonstrated increased CD4 + T and CD19 + B cell numbers. Treatment also resolved many clinical and lab complications of ADA deficiency such as depleted lymphocyte subset counts, low ADA activity, hypogammaglobulinemia, infections, weight loss etc. Patients that had transitioned from pegademase to elapegademase had no new adverse effects.

Here we report real-world experience from the elapegademase registry (NCT03878069), a single arm, open-label, multicenter registry study in patients with ADA-SCID who required ERT treatment with elapegademase. The registry was carried out as a post-marketing commitment [22], and started shortly after the approval of elapegademase in the U.S. by the FDA. The first patient was enrolled on 30 Sep 2019; patients were followed for at least two years or until they had undergone allogeneic HSCT. The study completed on 18 Jan 2023.

## Methods

### Study Design

This was a single arm, open-label, multicenter registry study in patients with ADA-SCID who required enzyme replacement therapy (ERT) treatment with elapegademase. The study, including assessments, was conducted under conditions of routine clinical care. All patients received elapegademase, and therefore blinding was not applicable. Due to the registry nature of this study, there was no pre-determined sample size. Recruitment continued until the number of patients enrolled was deemed to be sufficient to generate a meaningful amount of data on the safety and effectiveness of elapegademase.

### Study Population

Eligible patients for enrollment included: (1) patients who received long-term ERT with pegademase or elapegademase (as part of the Phase 3 clinical trial (STP-2279-002), previously on pegademase); (2) ERT-naïve infants diagnosed via newborn screening and definitive testing for ADA deficiency and prescribed elapegademase; (3) patients who received elapegademase while preparing for HSCT or HSC-GT; and (4) those who declined, were ineligible for, or did not respond to HSCT or HSC-GT and who resumed/initiated elapegademase therapy. Individuals who had any condition that in the opinion of the treating physician made them unsuitable for the study were excluded from participation.

For analyses, patients were divided into three groups based on their previous experience with ERT: (1) ERT-naïve patients (EN; infants and children with no prior experience with ERT); (2) pegademase-transitioning patients (PT; patients who had been taking pegademase for varying durations of time before switching to elapegademase); (3) STP-2279-002 patients (STP; included those who had taken part in the U.S. Phase 3 trial [16]). Patients in the STP group were also pegademase-transitioning as they had been on pegademase prior to enrolling in the trial and had continued it for a few weeks before switching to elapegademase. Compared to the PT group, STP patients had been on elapegademase for longer, and they had been followed prior to their enrollment in the registry.

### Assessments and Dose Administration

Suggested monitoring recommendation and schedule of assessments was provided based on the United States Prescribing Information (USPI) for elapegademase [15]. The optimal long-term dose and the schedule of administration were established by the treating physician for each patient individually. In the study, patients self-administered; or had a caregiver or healthcare provider (HCP) administer an intramuscular (IM) dose of elapegademase on the dosage and schedule recommended by the treating physician. Unlike a clinical trial, the sites did not collect used vials to verify whether the medication had been taken, and commercially available elapegademase was used. Compliance could therefore not be assessed.

If a patient had been receiving pegademase at a dose of ≤ 30 U/kg/week, or if information on pegademase dosing was not available, the starting weekly dose of elapegademase was 0.2 mg/kg. If a patient had been receiving pegademase at a dose of > 30 U/kg/week, an equivalent elapegademase dose (mg/kg) was calculated using the following equation: *Elapegademase dose in mg/kg = (Pegademase dose in U/kg)/(150)*. In both cases, the dose could be increased by increments of 0.033 mg/kg weekly if trough ADA activity was under the optimal threshold of 30 mmol/h/L, trough dAXP concentration was above the detoxified threshold of 0.02 mmol/L, and/or the immune reconstitution was inadequate based on clinical assessment. The total weekly dose could be divided and administered in multiple IM injections per week. If a patient had never previously received pegademase, the starting weekly dose with elapegademase was 0.4 mg/kg of body weight, divided into two weekly doses, for a minimum of 12 to 24 weeks until immune reconstitution was achieved based on clinical assessment by the treating physician.

Patients in the EN group needed to be followed more frequently over the first year than those who had previously received pegademase. Patients in the STP group did not need to have retrospective data collected on their first 6 months of elapegademase treatment, as this was previously analyzed [16]. The frequency of monitoring was to be increased if therapy was interrupted or if an enhanced rate of clearance of plasma ADA activity developed. For patients who received HSCT or HSC-GT, safety outcomes and survival were assessed at 1 and 6 months after the last elapegademase dose.

### Data Collection and Definitions

The data, whether collected prospectively within the registry study or retrospectively from earlier records, included effectiveness variables (trough plasma ADA activity, total trough erythrocyte dAXP, immune status [lymphocyte subsets], clinical status [infections, hospitalizations, and overall survival]), and safety variables (adverse events, safety laboratory assessments, physical examination, vital signs, immunogenicity, and concomitant medications). A yearly review of the study data was conducted by a Data Safety Monitoring Committee (DSMC) to identify any safety signals or trends. The biochemical analyses were used both to provide information on safety and effectiveness measures and to determine the need for dose adjustments. Due to the registry nature of this study, the time points at which variables were measured were not standardized as in a clinical trial. Consequently, certain data points collected were limited and sparse.

Patients who received HSCT or HSC-GT could discontinue ERT, although they were asked to return on two occasions for safety follow-ups. All other patients were followed for a minimum of 24 months of treatment, including treatment that had taken place prior to enrollment.

All adverse events (AEs) that were deemed at least possibly related to the administration of elapegademase, along with clinically meaningful laboratory abnormalities, were reported. Additionally, infections and any AEs that led to hospitalization were also reported, irrespective of causal relationship to elapegademase.

Immunogenicity was assessed as required. If trough plasma ADA activity fell to below 15 mmol/h/L during elapegademase treatment, anti-drug antibodies should be suspected. Venous blood samples were collected in 2 mL ethylenediaminetetraacetic acid (EDTA) tubes to test for anti-elapegademase binding, neutralizing antibodies, and anti-PEG antibodies.

Baseline was defined as the last measurement of a variable before the first dose of elapegademase, whether the first dose was before or after enrollment in the registry. For EN patients, the baseline assessments took place after enrollment and all data were collected prospectively. All but one of the EN patients first had ADA levels measured after the start of elapegademase treatment, representing the early effects of treatment rather than actual pre-treatment status. For PT and STP patients, baseline was defined as the last available value prior to the initiation of elapegademase. For STP patients, baseline was measured in the previous Phase 3 clinical trial [16], with values being quantified at a different laboratory from that used in this registry. The earliest exposure to elapegademase was 10 Feb 2015. Overall, the first measure in this registry was not necessarily the baseline value (i.e., if obtained after elapegademase treatment began) and the last measure was not necessarily the end-of-study value (i.e., if obtained before the patient ended study participation).

### Statistical Analyses

Given the nature of this study, all analyses were descriptive, and no statistical hypotheses were tested. 95% confidence intervals (CIs) were calculated for the main outcome variables without adjustment for multiplicity.

### Ethics

The study protocol was reviewed by the Independent Ethics Committee or Institutional Review Board for each center. Informed written consent was obtained from each patient before collecting any data. Additional information on the study was provided verbally by the study investigator or in a written format. The study was conducted in accordance with the principles laid down in the Declaration of Helsinki and Good Clinical Practice [23, 24].

## Results

### Patient Characteristics

**Fig. 1:**
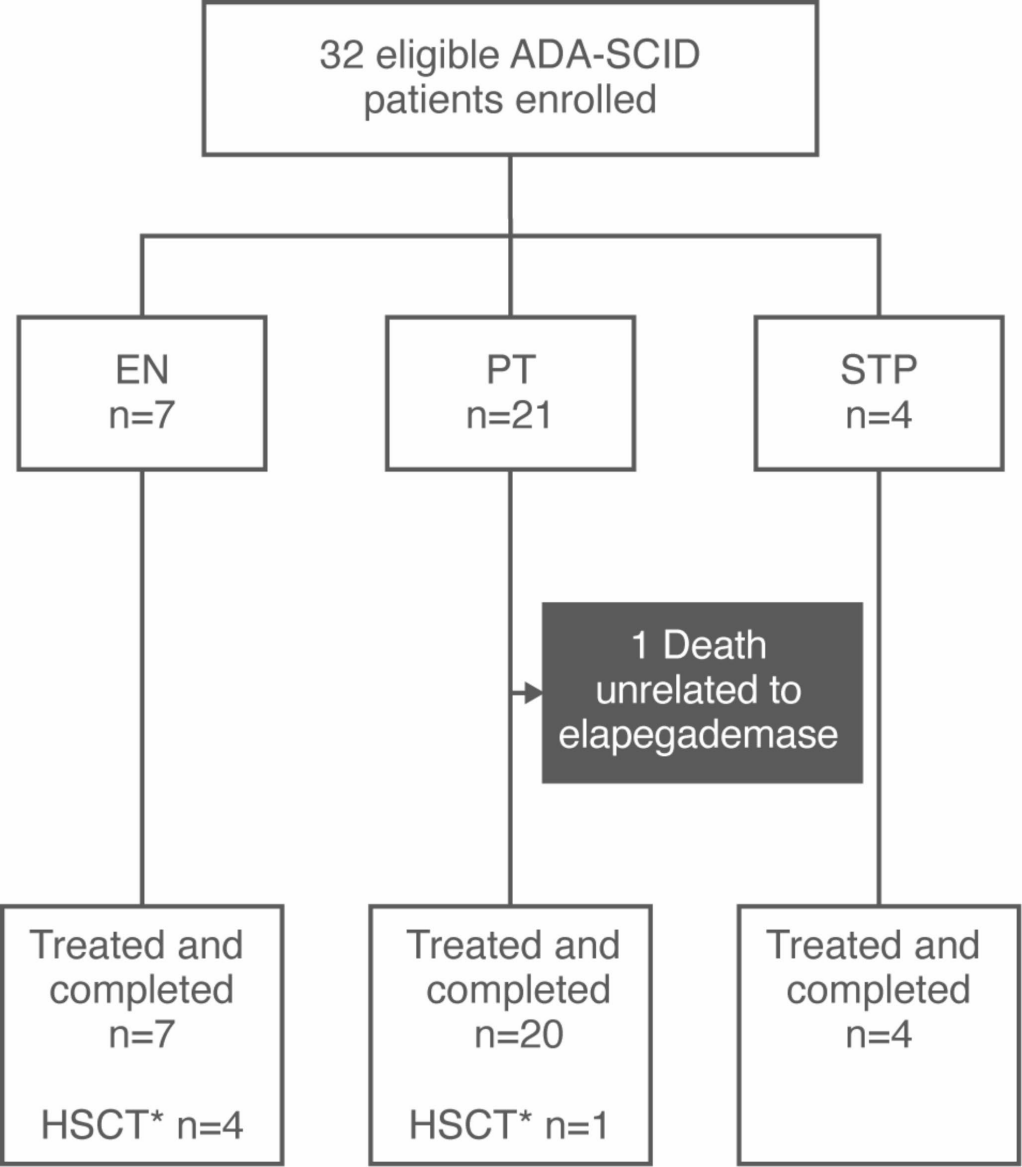
Patient disposition. ADA-SCID, adenosine deaminase deficient severe combined immunodeficiency; HSCT, hematopoietic stem cell transplant​ *These patients underwent HSCT

The study included 32 patients from 14 U.S. sites (Table 1; Fig.S1). All but one patient (96.9%) completed the study: 5 patients remained until they underwent HSCT, and 26 remained until the study completion (Fig. 1). The single patient in the PT group who did not complete the study died shortly after being hospitalized for severe pneumonia and respiratory failure unrelated to elapegademase treatment (Supplemental Text: Individual Patient Narratives; duration of elapegademase treatment: 11 months).

All patients had one or two weekly doses of elapegademase, except Patient 20 who had three weekly doses at one timepoint. Dosing dates, times, and volumes were self-reported or reported by HCPs and could not be verified. There were no reports of omissions or delays in the EN and STP groups. In the PT group, 4 patients reported missing or being late for at least one dose; 87.5% of all patients did not report dose omissions or delays.

Most patients were ADA-SCID patients, diagnosed between 6 and 12 months (median age at diagnosis 0.2 years). Three patients in the PT group were late-onset ADA-CID patients (Table S1); two patients had homozygous missense mutation in ADA exon 6, V177M [C.529G > A]), a homozygous missense mutation in ADA exon 6, a previously identified change shown in vitro to produce 0.1–0.4% of wild type ADA activity [9].

Patients in the PT group received pegademase for a mean of 154.7 months (slightly less than 13 years), with a range of 2.1–390.1 months (32.5 years). Patients in the STP group received pegademase from 13 to 25 years before the Phase 3 trial [16] and a further 1.2 months after the trial began and before being transitioned to elapegademase.

**Table 1:** Patient demographics

		EN (*n* = 7)	PT (*n* = 21)	STP (*n* = 4)	Overall (*N* = 32)
**Age at first diagnosis of ADA deficiency (years)**	Mean ± SD	0.03 ± 0.03	2.8 ± 4.9	1.9 ± 2.2	2.1 ± 4.1
	Median	0.02	0.5	1.1	0.2
	Min, Max	0, 0.09	0, 16.9	0, 5.0	0, 16.9
**Age at initiation** **of elapegademase** **(years)**	Mean ± SD	0.9 ± 0.6	19.7 ± 12.1	25.5 ± 6.9	16.3 ± 13.1
	Median	1.0	19.0	23.5	18.5
	Min, Max	0, 2	2, 47	20, 35	0, 47
Sex*,* n*** (%)**	Female	3 (42.9)	15 (71.4)	2 (50.0)	20 (62.5)
	Male	4 (57.1)	6 (28.6)	2 (50.0)	12 (37.5)
**Self-identified race**, *n* **(%)**	White	4 (57.1)	10 (47.6)	4 (100)	18 (56.3)
	Black	1 (14.3)	8 (38.1)	-	9 (28.1)
	Asian	0	1 (4.8)	0	1 (3.1)
	Other	1 (14.3)	0	0	1 (3.1)
	Multiple	1 (14.3)	2 (9.5)	-	3 (9.4)
**Self-identified ** **ethnicity**,* n*** (%)**	Hispanic or Latino	2 (28.6)	3 (14.3)	2 (50.0)	7 (21.9)
**Any prior non-ERT treatments**,* n*** (%)**	Yes	-	4 (19.0)	1 (25.0)	5 (15.6)
	No	7 (100.0)	16 (76.2)	2 (50.0)	25 (78.1)
HSCT prior treatment**, *n* (%)		-	2 (9.5)	-	2 (6.3)
HSC-GT prior treatment, *n* (%)		-	3 (14.3)	1 (25.0)	4 (12.5)
**Duration of elapegademase treatment** ^**a**^	Mean (SD), months	16.4 (11.5)	36.8 (12.2)	39.6 (10.6)	32.7 (14.5)
	Minimum-Maximum, months	1.6–30.5	11.9–48.6	23.7–45.4	1.6–48.6

ERT, enzyme replacement therapy; HSC-GT, hematopoietic stem cell gene therapy; HSCT, hematopoietic stem cell transplant SD, standard deviation

*Collected as male or female. **One patient received both HSCT (1999) and HSC-GT (2003) ^a^Duration of treatment is calculated as (last day of treatment (or data cut-off if ongoing) – first day of treatment + 1 day)/30.4375. The first day of the month was used in the case of partial dates. One month = 30.4375 days

### Effectiveness

#### Trough Plasma ADA and Total Trough Erythrocyte dAXP

**Fig. 2:**
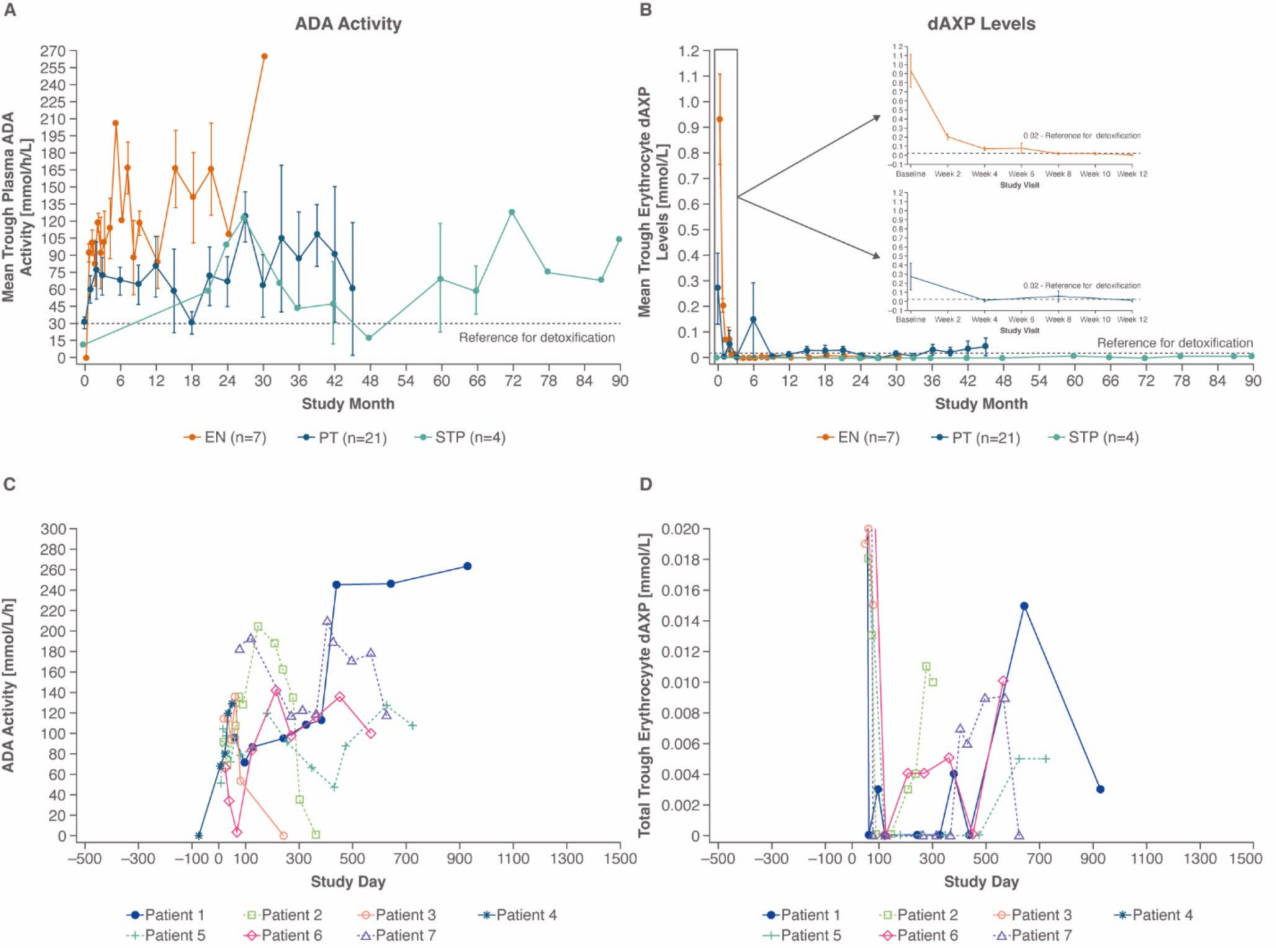
Mean ADA activity and dAXP levels over time. **A** and **B**: The mean and standard error (SE) for ADA activity and plasma dAXP for all time points were plotted by patient group. Cut-off values for meeting full metabolic detoxification are indicated by the horizontal line. **C** and **D**: EN group individual patient ADA activity and dAXP is plotted over time

By the end of the study, most patients (*n* = 24; 75%) attained or maintained values that met or exceeded the minimum threshold for plasma ADA (≥ 30 mmol/h/L) and met or were below the maximum threshold for dAXP (≤ 0.02 mmol/L) (Fig. 2A-B). Most significant improvements regarding ADA and dAXP were seen in the youngest EN patients (Fig. 2C-D). Most EN patients (*n* = 6; 85.7%) met both ADA and dAXP thresholds at final measurement. Patient 4 had increased ADA activity and a decreased dAXP level following 1.6 months of elapegademase treatment, but his dAXP level was still above the toxic threshold at the final measurement before undergoing HSCT​ (Table S2).

All STP patients and most PT patients (*n* = 14; 66.7%) continued to maintain satisfactory levels of both ADA and dAXP (Fig. 2; Fig. S2). Some patients (*n* = 8; 25%) did not attain or maintain both optimal ADA levels and detoxified dAXP levels by the end of the study (Table S2): 5 patients had toxic levels of dAXP, while 3 patients had sub-optimal levels of ADA, but had detoxified levels of dAXP.

Some individuals (*n* = 5; 15.6%) had failed HSCT or HSC-GT and resumed ERT treatment​ as a life-sustaining therapy (Table 2). Three of the patients had met the ADA and dAXP thresholds for detoxification by the end of the study.

**Table 2:** Patient characteristics of those who failed previous non-ERT therapy

Patient ID	Group	Previous non-ERT therapy	Previous non-ERT therapy date	ERT Pre-treatment/ total weekly dose	ERT Pre-treatment start date	ERT Pre-treatment stop date	Result of prior non-ERT treatment	Date of elapegademase initiation	Elapegademase treatment regimen	Total lymphocyte count (10^9^/L) at last timepoint*	ADA (mmol/h/L) (optimal threshold ≥ 30 mmol/h/L) at last timepoint	dAXP (mmol/L) (toxicity threshold ≤ 0.02 mmol/L) at last timepoint
9	PT	Gene Therapy - retroviral	1993	Pegademase/ 2250U	1993	2019-03	Failure	2019-03	8.4 mg 2x/week	0.3	*16.7*	*0.03*
13	PT	HSCT - haploidentical Mother	1997	Pegademase/ 1200U	2017-07	2018-04	Partial immune reconstitution	2019-04	14.4 mg 2x/week	0.3	*4.6*	*0.02*
14	PT	HSCT – haploidentical Father	1999	Pegademase/ 750U	2017-11	2019-04	Failure	2019-05	4.8 mg 1x/week	1.2	**37.1**	**0.0**
		Gene Therapy – retroviral	2001-03 2003-10				Partial immune reconstitution Failure					
18	PT	Gene Therapy - retroviral	1990	Pegademase/ 750U	1990-09	2019-03	Failure	2019-03	14 mg 1x/week	0.9	**149.6**	**0.006**
32	STP	Gene Therapy - retroviral	2001	Pegademase/ 1500U	2016-11	2017-01	Failure	2019-05	12 mg 1x/week	0.7	**57.6**	**0.003**
				Elapegademase/ 10 mg	2017-01	2019-05						

*All patients had lymphocyte levels below reference ranges. Key: *below threshold for detoxification*; **meets threshold for detoxification**

#### Levels of Lymphocytes

**Fig. 3:**
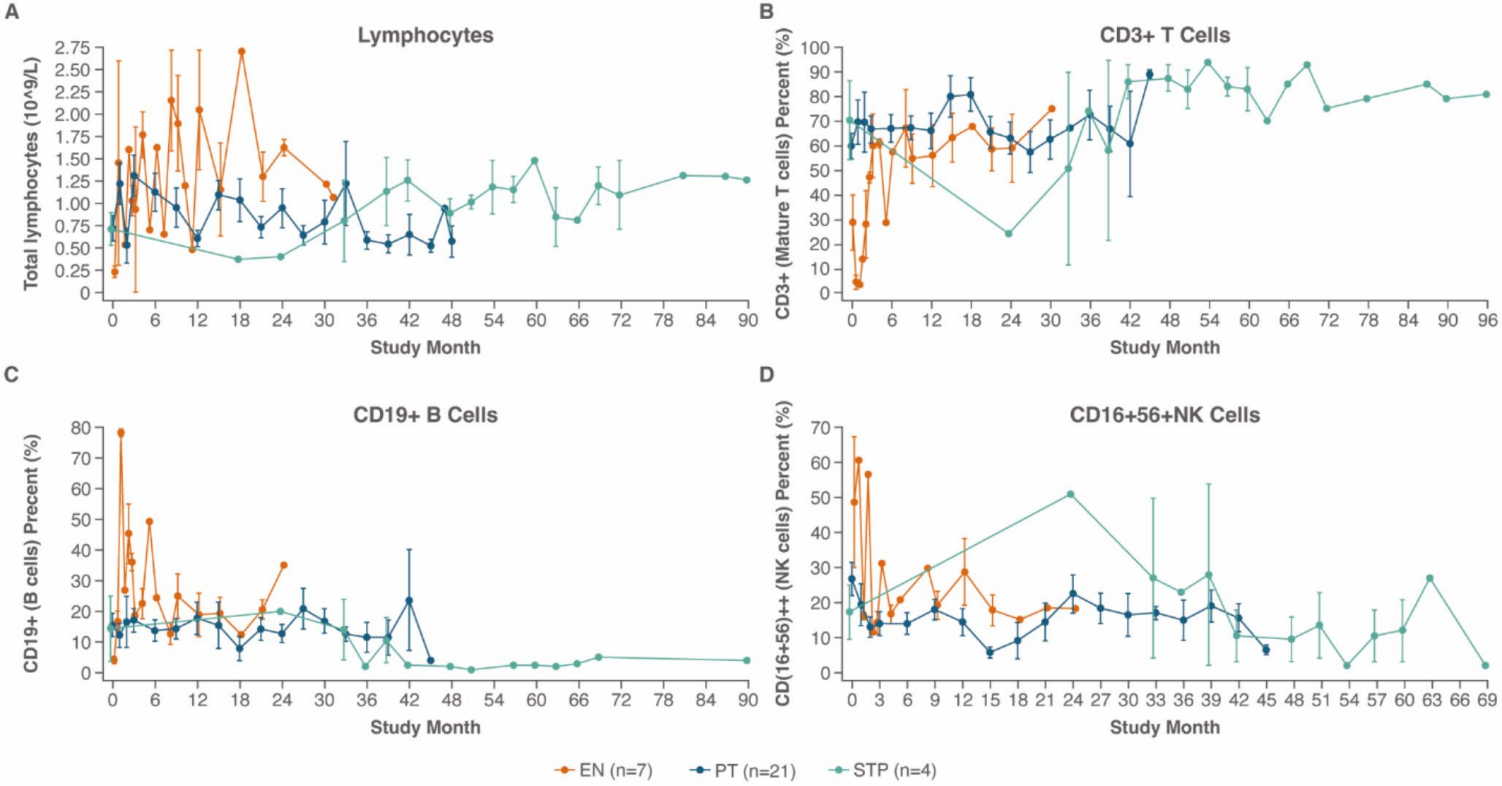
Mean total lymphocytes and mean percentage of cell subsets over time. The mean and standard error (SE) for all time points was plotted by patient group. Sampling of **(A)** lymphocytes **(B)** mature T cells **(C)** B cells and **(D)** NK cells was inconsistent and sparse. No meaningful analyses could be performed

Only 9 patients (2 EN and 7 PT) had the first and last measurements of total lymphocyte counts, B-cells, T-cells, and NK cells done on the same dates. Accordingly, the findings are presented separately for each subset. Immunoglobin counts for IgA, IgM and IgG were collected; however, data were too sparse or inconsistent to draw meaningful conclusions. The EN group had higher lymphocyte counts than the other groups, (Fig. 3; **Fig. S3**)

#### Clinical Status

Infants born with ADA-SCID have impaired immunity at birth; 3 EN patients experienced infections during the first year of treatment (Table 3). For the PT and STP groups, the rates of infection remained constant. Rates of infections were lower in patients who met both the targeted ADA and dAXP thresholds (0.85) than in those who did not meet both thresholds (1.56). No consistent trend was seen in the rates of hospitalizations between patients who met both effectiveness thresholds versus those who did not.

Of the 88 treatment-emergent infections in 23 (71.9%) patients (Table 3), 22 in 12 (37.5%) patients were serious and required hospitalization. The most common infection was COVID-19 (18 infections in 11 (34.4%) patients), which emerged around the time the study began in late 2019 and was declared a pandemic shortly thereafter. Three patients were hospitalized because of COVID-19 infection and treated with intravenous remdesivir. One of these patients, Patient 7 (EN group), was additionally hospitalized at a different time for acute kidney injury, while the other two (Patients 8 and 9; PT group) were hospitalized only because of severe COVID-19, although they both had mild COVID-19 twice prior. No COVID-19 related death was reported during the study period and all COVID infections were resolved​.

A total of 27 hospitalizations occurred during the study in 15 (46.9%) patients (Table 3). The most common causes for hospitalizations were associated with events in the MedDRA system organ class (SOC) of infections and infestations followed by those in the respiratory, thoracic and mediastinal disorders SOC (Table 3; Table S3). Removing the effect of the COVID-19 pandemic, the 27 hospitalizations in 15 patients would have been 24 hospitalizations in 13 patients; about a 10% difference.

**Table 3:** Summary of infections and hospitalizations

	EN (*n* = 7)	PT (*n* = 21)	STP (*n* = 4)	Overall (*N* = 32)
**Patients with infections**,* n*** (%)**	4 (57.1)	16 (76.2)	3 (75.0)	23 (71.9)
**Total number of infections**,* n*	15	65	8	88
**Patients with hospitalizations**,* n*** (%)**	2 (28.6)	11 (52.4)	2 (50.0)	15 (46.9)
**Total number of hospitalizations**,* n*	5	17	5	27
**Duration of hospitalization**, **months**,** mean (SD)**	0.2 (0.1)	0.4 (0.4)	1.1 (0.6)	0.5 (0.5)

SD, standard deviation

### Safety

No new, unexpected safety concerns occurred in this study. No related AEs were seen in the STP patients, who had been on elapegademase the longest (Table 4). Of 117 treatment-emergent AEs (TEAEs) in 26 patients (Table S3), just 7 events in 4 (12.5%) patients were deemed to be at least possibly related to elapegademase (Table 4). Of these 4 patients, 3 were in the EN group; thrombocytosis, hypercalcemia, and neutropenia were observed while on elapegademase, but treatment did not need to be discontinued. The most reported TEAEs were infections (24 [75.0%]); respiratory, thoracic, and mediastinal disorders (7 [21.9%]); and blood and lymphatic system disorders (6 [18.8%]).

Nineteen patients (59.4%) experienced a total of 39 serious AEs (SAEs); none were related to elapegademase treatment. Most events (*n* = 26; 67%) were in the SOC of infections and infestations, followed by 6 events in the SOC of respiratory, thoracic and mediastinal disorders, and 2 events in the SOC of gastrointestinal disorders. No more than one event was reported for any other SOC. Pneumonia and bronchiectasis in 2 patients resolved with sequelae and were ongoing at the time of database lock, one patient died, and all other SAEs resolved. The most common event (11 [34.4%]) was infection with COVID-19.

The protocol recommended to test for anti-elapegademase binding and neutralizing antibodies and anti-PEG antibodies if a patient showed a persistent decrease in trough plasma ADA activity to below 15 mmol/h/L. Testing was done on only 2 patients (with negative results).

**Table 4:** Summary of related adverse events

	EN (*n* = 7)	PT (*n* = 21)	STP (*n* = 4)	Overall (*N* = 32)
**Patients with at least one related adverse event**	3 (42.9)	1 (4.8)	0	4 (12.5)
Thrombocytosis	2 (28.6)	0	0	2 (6.3)
Neutropenia	1 (14.3)	0	0	1 (3.1)
Injection site erythema	0	1 (4.8)	0	1 (3.1)
Exposure via skin contact	0	1 (4.8)	0	1 (3.1)
Weight increased	0	1 (4.8)	0	1 (3.1)
Hypercalcemia	1 (14.3)	0	0	1 (3.1)

All events resolved without sequelae except for weight gain, for which the outcome was unknown

## Discussion

In alignment with Phase 3 clinical studies on elapegademase [16, 21], most patients in this registry attained or maintained ADA activity and dAXP values that met or surpassed the defined detoxification thresholds. As a registry study, patients received routine clinical care, and HCPs had discretion over the frequency and timing of visits. Although efforts were made to record dosing dates, times, and volumes on medication administration forms, compliance data were ultimately self-reported by patients or by HCPs and could not be verified as in a clinical trial. In patients with enough compliance data recorded, ADA and dAXP levels aligned with treatment compliance (e.g., missed doses led to decreased ADA activity and increased dAXP levels). In patients that did not maintain satisfactory ADA and dAXP levels, inconsistency in dosage administration in some patients, as well as lack of adherence was the underlying reason.

Recent real-world data on infants with ADA-SCID [17, 18], a key patient group treated with elapegademase for which limited data exist, echo these findings. The EN group, who began elapegademase within weeks of birth without prior definitive treatment, maintained optimal plasma ADA activity, increased lymphocyte counts, had manageable infections, and had no mortality for up to 30 months while on elapegademase. Like findings with pegademase, initiation of elapegademase rapidly increased plasma ADA activity and sharply reduced dAXP to nearly undetectable levels [18]. In these infants, mean and median trough plasma ADA activity has been shown to range from 100 to 165 µmol/mL [17], levels 1.5 to 2.5-fold higher than trough levels reported in patients who received comparable doses of pegademase and in patients from the U.S. clinical trial of elapegademase [16, 25, 26]. Infants in this study had mean trough plasma ADA levels ranging from 83.2 to 264.2 µmol/mL, which was generally well tolerated except for two cases of thrombocytosis, one case of neutropenia and one case of hypercalcemia. Thrombocytosis has been observed in some patients on ADA ERT [15, 17, 27, 28, 29], and the mechanism remains unclear. None of the patients in this registry or in other studies of ADA ERT suffered adverse sequelae related to thrombocytosis, nor did they need to discontinue ERT. The highest mean trough plasma ADA level in the PT group was 124.4 µmol/mL and 128.6 µmol/mL in the STP group.

Long-term ERT use has documented drawbacks, including risks of malignancy, partial immune reconstitution or dysregulation, immunogenicity, and decreased lymphocyte function [14, 19, 30]. Patients in the STP group, treated up to nearly 8 years, were detoxified at both time points, indicating the long-term effectiveness of elapegademase. Lymphocyte levels were also maintained in this group over the course of the study. Although data on the immune cell subsets were too limited for definitive conclusions, a trend towards steady levels of T and B cells across all patients was observed, contrasting with reports of declining immune cell counts over time with pegademase [19, 31]. In general, patients in this registry had lymphocyte levels that were below or near the low end of age-adjusted reference ranges at all time points measured, yet life-threatening infections were prevented, and overall health was maintained. Consistent with previous reports [17, 18], lymphocyte counts increased in the EN group, while the results were more mixed in patients previously treated with ERT.

Fluctuations in ADA activity were observed across all groups during the study, and previous reports have shown that neutralizing antibodies can decrease efficacy of ADA ERT [3, 25, 27, 32]. Since testing for immunogenicity was not routinely conducted in this registry, it seems that most HCPs did not find it necessary, given the overall clinical status with mostly manageable infections and dAXP levels within the detoxified range. While it cannot be confirmed that no patients developed anti-drug antibodies, the majority achieved detoxification, suggesting immunogenicity was not a significant issue.

During the registry period, the COVID-19 pandemic significantly impacted global health, leading to unique circumstances where many AEs were related to COVID-19 infections and respiratory complications; COVID-19 was the most frequently reported infection. The pandemic indirectly affected the number of AEs through delays in elective procedures and follow-up care [33]. Despite challenges caused by the pandemic, there were no COVID-19 related deaths. Safety data were not collected in a manner conducive to comprehensive summaries over time; however, overall tolerance of elapegademase was consistent with previous real-world and clinical studies [16, 17, 18, 21].

## Conclusions

This registry study demonstrates that ERT-naïve infants and children with ADA-SCID (EN group) attained and maintained optimal plasma ADA activity, increased lymphocyte counts, and had manageable infection rates with no mortality while being treated with elapegademase for up to 30 months. Patients previously enrolled in the US Phase 3 study (STP group) and 66.7% of those transitioning from pegademase (PT group) also maintained satisfactory ADA and dAXP levels with stable rates of infections, hospitalizations, and lymphocyte counts for up to 48.6 months. Despite some variability in measures, these patients did not experience clinical deterioration (no increase in rates of infections and hospitalizations) over the treatment period. Elapegademase showed long-term effectiveness and safety, up to 4 years, highlighting its role in maintaining immunity and preventing severe infections. Limitations of the study include sparse and inconsistent data collection on immune function, immunoglobulin levels, growth, quality of life, and immunogenicity, which suggests areas for future research.

A plain language summary of this study is available as supplemental material (Supplemental Text: Real-World Experience with Elapegademase for ADA-SCID).

## Electronic Supplementary Material

Below is the link to the electronic supplementary material.

Supplementary Material 1

## Data Availability

All data required to evaluate the conclusions in the paper are present in the manuscript or its appendix. Further information on the study protocol or de-identified datasets generated and analyzed within this publication are available from the corresponding author on reasonable request.

## References

[1] Flinn AM, Gennery AR. Adenosine deaminase deficiency: a review. Orphanet J Rare Dis. 2018;13(1):65. 29690908 10.1186/s13023-018-0807-5 PMC5916829

[2] Gaspar HB, Aiuti A, Porta F, Candotti F, Hershfield MS, Notarangelo LD. How I treat ADA deficiency. Blood. 2009;114(17):3524–32. 19638621 10.1182/blood-2009-06-189209 PMC2766674

[3] Hershfield M. Adenosine deaminase deficiency. Adam MP FJ, Mirzaa GM, et al., editors. Seattle (WA): University of Washington, Seattle; 1993–2024. 2006 Oct 3 [Updated 2024 Mar 7]. 20301656

[4] Hershfield MS, IMMUNODEFICIENCY CAUSED BY ADENOSINE, DEAMINASE DEFICIENCY. Radiol Clin North Am. 2000;20(1):161–75.

[5] Gaspar HB. Bone marrow transplantation and alternatives for adenosine deaminase deficiency. Immunol Allergy Clin North Am. 2010;30(2):221–36. 20493398 10.1016/j.iac.2010.01.002

[6] Bradford KL, Moretti FA, Carbonaro-Sarracino DA, Gaspar HB, Kohn DB. Adenosine deaminase (ADA)-Deficient severe combined immune deficiency (SCID): molecular pathogenesis and clinical manifestations. J Clin Immunol. 2017;37(7):626–37. 28842866 10.1007/s10875-017-0433-3

[7] Cicalese MP, Ferrua F, Castagnaro L, Pajno R, Barzaghi F, Giannelli S, et al. Update on the safety and efficacy of retroviral gene therapy for immunodeficiency due to adenosine deaminase deficiency. Blood. 2016;128(1):45–54. 27129325 10.1182/blood-2016-01-688226 PMC5325048

[8] Whitmore KV, Gaspar HB. Adenosine deaminase deficiency - More than just an immunodeficiency. Front Immunol. 2016;7:314. 27579027 10.3389/fimmu.2016.00314 PMC4985714

[9] Arredondo-Vega FX, Santisteban I, Daniels S, Toutain S, Hershfield MS. Adenosine deaminase deficiency: genotype-phenotype correlations based on expressed activity of 29 mutant alleles. Am J Hum Genet. 1998;63(4):1049–59. 9758612 10.1086/302054 PMC1377486

[10] Hershfield MS. Genotype is an important determinant of phenotype in adenosine deaminase deficiency. Curr Opin Immunol. 2003;15(5):571–7. 14499267 10.1016/s0952-7915(03)00104-3

[11] Kuo CY, Garabedian E, Puck J, Cowan MJ, Sullivan KE, Buckley RH, et al. Adenosine deaminase (ADA)-Deficient severe combined immune deficiency (SCID) in the US immunodeficiency network (USIDNet) registry. J Clin Immunol. 2020;40(8):1124–31. 32880085 10.1007/s10875-020-00857-9 PMC8216639

[12] Kohn DB, Hershfield MS, Puck JM, Aiuti A, Blincoe A, Gaspar HB, et al. Consensus approach for the management of severe combined immune deficiency caused by adenosine deaminase deficiency. J Allergy Clin Immunol. 2019;143(3):852–63. 30194989 10.1016/j.jaci.2018.08.024 PMC6688493

[13] Adenosine deaminase deficiency: National Institutes of Health - Genetic and Rare Diseases Information Center. [Available from: https://rarediseases.info.nih.gov/diseases/5748/adenosine-deaminase-deficiency

[14] Grunebaum E, Booth C, Cuvelier GDE, Loves R, Aiuti A, Kohn DB. Updated management guidelines for adenosine deaminase deficiency. J Allergy Clin Immunol Pract. 2023;11(6):1665–75. 36736952 10.1016/j.jaip.2023.01.032

[15] Chiesi. REVCOVI (elapegademase-lvlr) injection for intramuscular use. Prescribing Information; December 2020.

[16] Dorsey MJ, Rubinstein A, Lehman H, Fausnight T, Wiley JM, Haddad E. PEGylated Recombinant adenosine deaminase maintains detoxification and lymphocyte counts in patients with ADA-SCID. J Clin Immunol. 2023;43(5):951–64. 36840835 10.1007/s10875-022-01426-y PMC10276086

[17] Hicks ED, Hall G, Hershfield MS, Tarrant TK, Bali P, Sleasman JW, et al. Treatment with Elapegademase restores immunity in infants with adenosine deaminase deficient severe combined immunodeficiency. J Clin Immunol. 2024;44(5):107. 38676811 10.1007/s10875-024-01710-z PMC11055758

[18] Murguia-Favela L, Suresh S, Wright NAM, Alvi S, Tehseen S, Hernandez-Trujillo V, et al. Long-term immune reconstitution in ADA-deficient patients treated with Elapegademase: A real-world experience. J Allergy Clin Immunol Pract. 2023;11(6):1725–33. 36736953 10.1016/j.jaip.2023.01.028

[19] Chan B, Wara D, Bastian J, Hershfield MS, Bohnsack J, Azen CG, et al. Long-term efficacy of enzyme replacement therapy for adenosine deaminase (ADA)-deficient severe combined immunodeficiency (SCID). Clin Immunol. 2005;117(2):133–43. 16112907 10.1016/j.clim.2005.07.006

[20] Turecek PL, Bossard MJ, Schoetens F, Ivens IA. PEGylation of biopharmaceuticals: A review of chemistry and nonclinical safety information of approved drugs. J Pharm Sci. 2016;105(2):460–75. 26869412 10.1016/j.xphs.2015.11.015

[21] Onodera M, Uchiyama T, Ariga T, Yamada M, Miyamura T, Arizono H, Morio T. Safety and efficacy of Elapegademase in patients with adenosine deaminase deficiency: A multicenter, open-label, single-arm, phase 3, and postmarketing clinical study. Immun Inflamm Dis. 2023;11(7):e917. 37506145 10.1002/iid3.917 PMC10367445

[22] Administration UFaD. [Available from: https://www.accessdata.fda.gov/drugsatfda_docs/appletter/2018/761092Orig1s000ltr.pdf

[23] ICH harmonised tripartite guideline. Guideline for good clinical practice. 8. Essential documents for the conduct of a clinical trial. J Postgrad Med. 2001;47(4):264–7. 11832645

[24] General Assembly of the World Medical A. World medical association declaration of Helsinki: ethical principles for medical research involving human subjects. J Am Coll Dent. 2014;81(3):14–8. 25951678

[25] Chaffee S, Mary A, Stiehm ER, Girault D, Fischer A, Hershfield MS. IgG antibody response to polyethylene glycol-modified adenosine deaminase in patients with adenosine deaminase deficiency. J Clin Invest. 1992;89(5):1643–51. 1569204 10.1172/JCI115761 PMC443041

[26] Weinberg K, Hershfield MS, Bastian J, Kohn D, Sender L, Parkman R, Lenarsky C. T lymphocyte ontogeny in adenosine deaminase-deficient severe combined immune deficiency after treatment with polyethylene glycol-modified adenosine deaminase. J Clin Invest. 1993;92(2):596–602. 8349799 10.1172/JCI116626 PMC294890

[27] Booth C, Gaspar HB. Pegademase bovine (PEG-ADA) for the treatment of infants and children with severe combined immunodeficiency (SCID). Biologics. 2009;3:349–58. 19707420 PMC2726071

[28] Booth C, Hershfield M, Notarangelo L, Buckley R, Hoenig M, Mahlaoui N, et al. Management options for adenosine deaminase deficiency; proceedings of the EBMT satellite workshop (Hamburg, March 2006). Clin Immunol. 2007;123(2):139–47. 17300989 10.1016/j.clim.2006.12.009

[29] Marwaha VR, Italia DH, Esper F, Hostoffer RW. Extreme thrombocytosis in response to PEG-ADA: early therapeutic and risk indicator. Clin Pediatr (Phila). 2000;39(3):183–6. 10752014 10.1177/000992280003900309

[30] Sauer AV, Brigida I, Carriglio N, Aiuti A. Autoimmune dysregulation and purine metabolism in adenosine deaminase deficiency. Front Immunol. 2012;3:265. 22969765 10.3389/fimmu.2012.00265 PMC3427915

[31] Malacarne F, Benicchi T, Notarangelo LD, Mori L, Parolini S, Caimi L, et al. Reduced thymic output, increased spontaneous apoptosis and oligoclonal B cells in polyethylene glycol-adenosine deaminase-treated patients. Eur J Immunol. 2005;35(11):3376–86. 16276484 10.1002/eji.200526248

[32] Mohamed F, Tarrant T, Hershfield M, Bali P, Pozos T. 128 Increased dosage of elapegademase-lvlr improved metabolic and Immunologic function in a patient with late-onset adenosine deaminase deficiency and neutralizing anti-drug antibodies. Clin Immunol. 2024;262:110070.

[33] Roy C, Bollman E, Carson L, Northrop A, Jackson E, Moresky R. Assessing the indirect effects of COVID-19 on healthcare delivery, utilization, and health outcomes: A scoping review. Eur J Pub Health. 2021;31. 10.1093/eurpub/ckab047 PMC8083627 33755130

