# Supplementary material for: Multi-Year Registry Study of Elapegademase Treatment in Patients With Adenosine Deaminase Severe Combined Immunodeficiency (ADA-SCID) Requiring Enzyme Replacement Therapy
Source: J Clin Immunol. 2025 Mar 27;45(1):84. doi: 10.1007/s10875-025-01873-3 (PMC11947034; doi:10.1007/s10875-025-01873-3)
Supplement: Supplementary file 1 — Supplementary Material 1 [file 10875_2025_1873_MOESM1_ESM.pdf]

Supplemental Figures

Figure 1. Registration sites

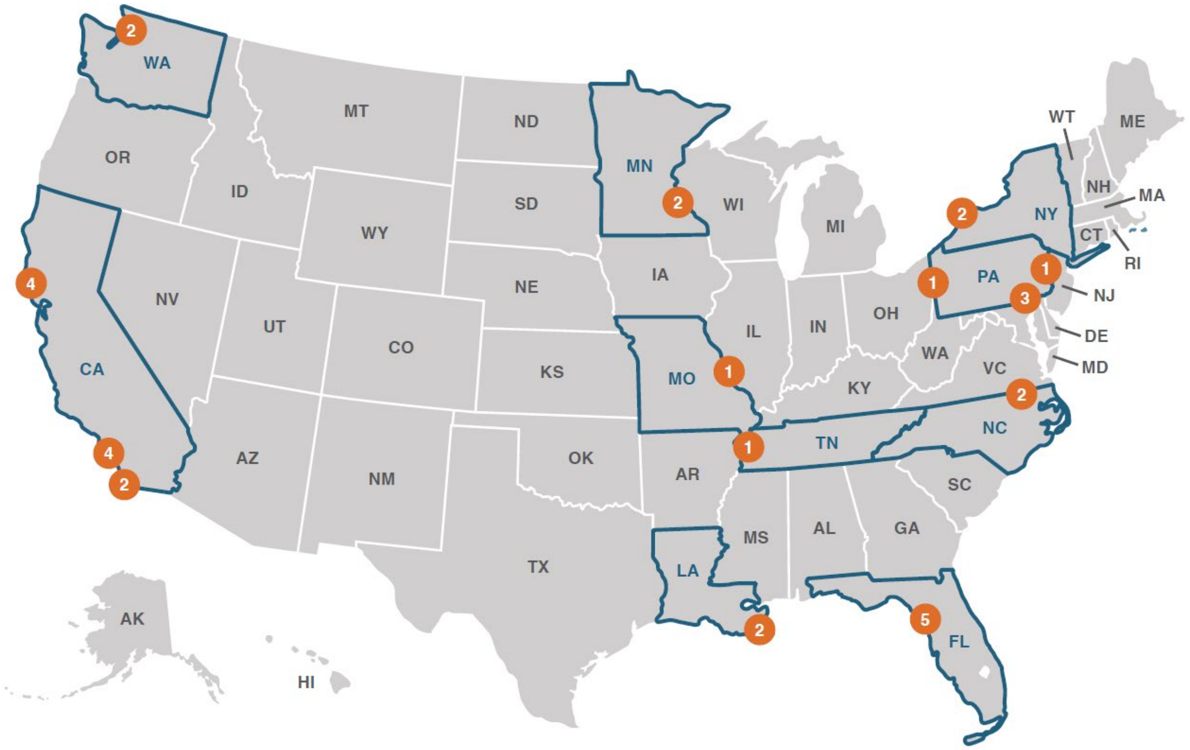

Figure 2. Individual plasma ADA levels and dAXP activity for PT and STP groups

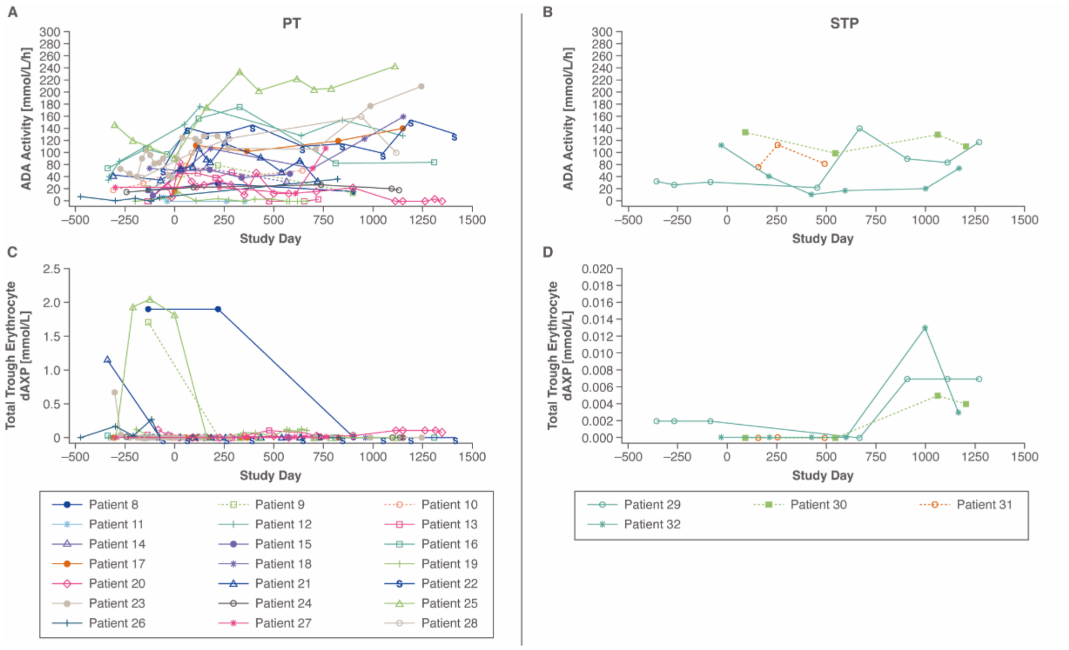

Figure 3. Individual ADA activity, trough erythrocyte dAXP levels, and total lymphocyte counts in late onset CID patients

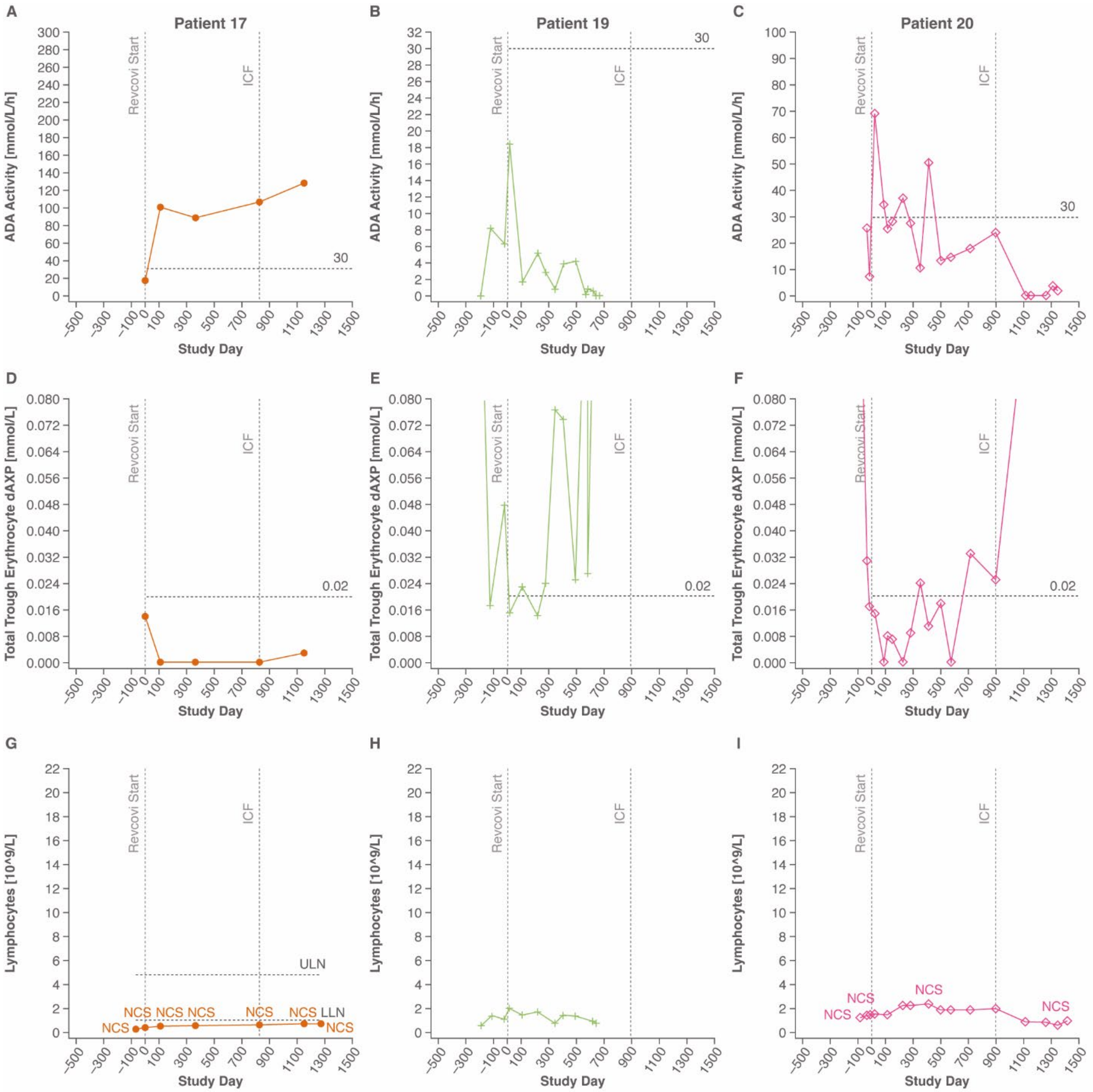

Figure 4. Individual total lymphocytes for patients in the EN group

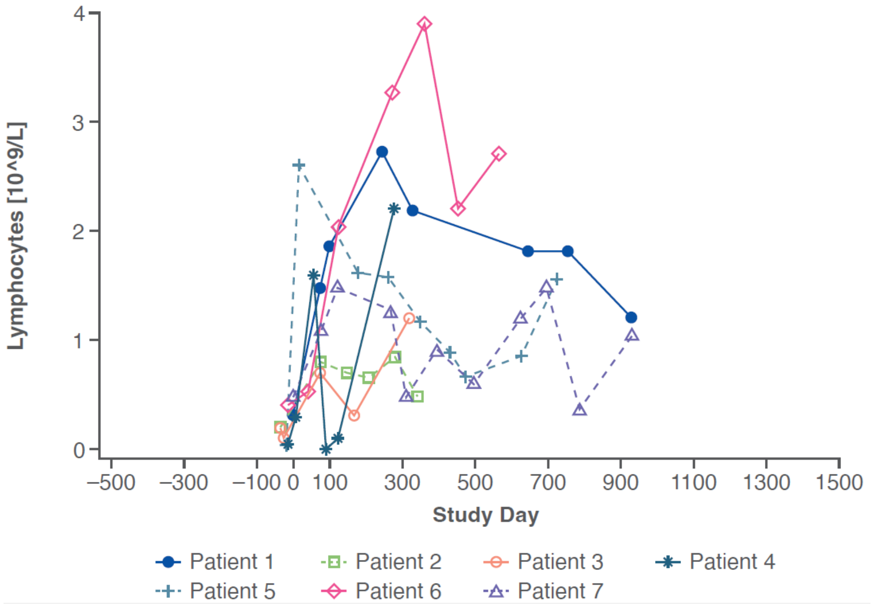

Supplemental Tables

Table 1 Patient demographics with age at diagnosis

Patient	Group	Age at diagnosis category	Age at diagnosis (years)	ADA Variant
1	EN	≤12 mths	0	C.320 T>C; C.632G>A heterozygous
2	EN	≤12 mths	0	C.956_960DEL (P.GLU319GLYFS*3); C.780+1DEL (splice site)
3	EN	≤12 mths	0	NA
4	EN	≤12 mths	0	NA
5	EN	≤12 mths	0	C956_960DEL (PGLU319GLYFS*3)
6	EN	≤12 mths	0	C.632 G>A; C.845 G>A
7	EN	≤12 mths	0	NA
8	PT	≤12 mths	0	Maternal G216R paternal deletion of promoter and exon 1
9	PT	≤12 mths	0	Maternal G216R paternal deletion of promoter and exon 1
10	PT	>12 mths–10 yrs	4	NA
11	PT	≤12 mths	0	NA
12	PT	≤12 mths	0	Homozygous for a pathologic ADA mutation
13	PT	≤12 mths	0	NA
14	PT	≤12 mths	0	NA
15	PT	>12 mths–10 yrs	2	NA
16	PT	≤12 mths	0	CHR20:G.43255158G>A (ARG101TRP); CHR: G.43251680C>T (GLY216ARG)
17	PT	> 10 yrs	12	C.646 G>A;

				C.467 G>A
18	PT	>12 mths–10 yrs	1	C.646G>A (P.GLY216ARG); C.478+6T>A
19	PT	> 10 yrs	16	Homozygous C.529G>A (V177M)
20	PT	> 10 yrs	11	Homozygous C.529G>A (V177M)
21	PT	≤12 mths	0	Homozygous C.320T>C (L107P)
22	PT	≤12 mths	0	Homozygous C.986C>T (ALA329VAL)
23	PT	≤12 mths	0	Homozygous C.986C<T (ALA329VAL)
24	PT	>12 mths–10 yrs	7	NA
25	PT	≤12 mths	0	NA
26	PT	≤12 mths	0	NA
27	PT	>12 mths–10 yrs	1	NA
28	PT	≤12 mths	0	NA
29	STP	≤12 mths	0	NA
30	STP	>12 mths–10 yrs	1	NA
31	STP	≤12 mths	0	NA
32	STP	>12 mths–10 yrs	5	NA

Key: Early onset SCID; delayed onset CID; late onset CID

NA, not available

Table 2. Patients who did not meet both thresholds for detoxification at the final timepoint

**Group**	**Patient ID**	**ADA (mmol/h/L) (Optimal threshold ≥30 mmol/h/L)**	**dAXP (mmol/L) (Toxicity threshold ≤ 0.02 mmol/L)**
EN	4	**130.8**	*0.22*
PT	8	*18.2*	**0.012**
PT	9	*16.7*	*0.026*
PT	11	*3.024*	**0.006**
PT	13	*4.57*	*0.023*
PT	19	*0*	*0.105*
PT	20	*1.76*	*0.082*
PT	24	*19.72*	**0.009**

Key: **Yes, meets the threshold for detoxification;** *No, does not meet the threshold for detoxfication*

Table 3. Summary of AEs

**Parameter**	**EN (n=7)**	**PT (n=21)**	**STP (n=4)**	**Overall (n=32)**
Total number of TEAEs	21	85	11	117
Incidence rate of TEAEs	2.13	1.32	0.83	1.34
Subjects with at least 1 TEAE, n (%)	5 (71.4)	18 (85.7)	3 (75.0)	26 (81.3)
Maximum severity of TEAEs:				
Mild, n (%)	1 (14.3)	5 (23.8)	1 (25.0)	7 (21.9)
Moderate, n (%)	1 (14.3)	9 (42.9)	1 (25.0)	11 (34.4)
Severe, n (%)	3 (42.9)	4 (19.0)	1 (25.0)	8 (25.0)
Total number of SAEs	12	22	5	39
Incidence rate of SAEs	1.22	0.34	0.38	0.45
Subjects with at least 1 SAE, n (%)	4 (57.1)	13 (61.9)	2 (50.0)	19 (59.4)

Supplemental Text

Individual Patient Narrative

Patient 11: This patient (black female; weight: 23 kg; age: 20–30 years) in the PT group received elapegademase 2.4 mg once a week throughout the study, with no dose changes or interruptions. She was hospitalized for pneumonia twice and once for acute hypercapnic respiratory failure. Approximately 11 months after the start of elapegademase treatment, she presented to the emergency room with Grade 3 (severe) pneumonia and respiratory failure. Testing showed that these events were unrelated to COVID-19. She additionally tested positive for rhinovirus and had leukopenia, anemia, and thrombocytopenia. A few days later, her dAXP level was detoxified at 0 mmol/L, but her ADA activity level was below the optimal threshold at 3.024 mmol/L/h. Her dose of elapegademase (based on body weight) was notably low, which might explain her ADA results. Due to her worsening condition, a decision was made by her family to have a Do Not Resuscitate (DNR) order put in place. The patient died, and in the view of the treating physician, her death was not related to a lack of effectiveness of elapegademase.

Real-World Experience with Elapegademase for ADA-SCID

Supplemental information to Dorsey MJ et al. J Clin Immunol

This is a summary of an article evaluating real world clinical outcomes for patients that were treated with elapegademase as a part of routine clinical care. This article was published in the *Journal of Clinical Immunology* in 2024.

How to say...

Elapegademase: "EL-uh-peg-AD-uh-mace"
Pegademase: "peg-AD-uh-mace"
Adenosine deaminase: "uh-DEH-nuh-seen dee-AH-muh-nays"

What is ADA-SCID?

ADA-SCID, also called **adenosine deaminase deficiency**, is a severe genetic disorder that impairs the development and function of immune cells called lymphocytes.

Lymphocytes are white blood cells that help the body fight infections. **A**denosine **dea**minase (ADA) is an enzyme that speeds up chemical reactions in the body. Those with ADA deficiency build up a substance called deoxyadenosine nucleotides (dAXP) in their cells, damaging the development of lymphocytes.

Untreated patients with ADA-SCID often develop chronic lung infections, chronic diarrhea, and widespread skin rashes. Affecting all races and sexes, this lifelong condition is typically diagnosed in young children.

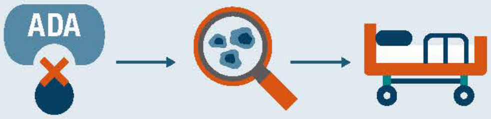

How is ADA-SCID Treated?

Enzyme replacement therapy (ERT) is a medicine that gives patients the ADA enzyme that the body can't make. This helps control the harmful substances that build up in cells due to the ADA deficiency, (dAXP). The ERT available in the U.S. for ADA-SCID treatment is elapegademase.

ERT is often used as a bridge therapy to keep a patient healthy until a **bone marrow transplant (BMT)**, also called **stem cell therapy (SCT)**, is possible. In some instances, **gene therapy (GT)** may be available. If these options are not possible, ERT can be continued. While a BMT or GT can replace the faulty ADA and restore the immune system; they do not work for everyone, so some patients still need ERT.

Why was the study done?

The study was performed to supplement limited clinical trial data. In this study, **real world data on the use of elapegademase** in a larger group of patients with ADA-SCID was collected.

Up to 2018, the only ERT available for ADA-SCID was **pegademase**. Researchers wanted to find out more about the safety and effectiveness of elapegademase in patients who had previously taken pegademase, and in newly diagnosed babies and children with ADA-SCID who had never been treated with ERT.

What did the researchers want to learn in this study?

1. Was elapegademase effective and safe in newly diagnosed babies and children?
2. Did switching from pegademase to elapegademase keep people healthy?
3. Did elapegademase restore lymphocyte levels?
4. What were the safety results of the study?

Who participated in the study

32 patients with ADA-SCID were treated with elapegademase at 14 medical centers in the U.S. from 2019 to 2023.

Elapegademase treatment duration
Average: 32.7 months

Age
Range: 0 to 47 years old
Average: 16 years old

Sex
62.5% Female (20 patients)
37.5% Male (12 patients)

Previous Treatments

7 had never received ERT **(referred to as the EN group [ERT-naïve])**

21 had previously received pegademase **(referred to as the PT group [pegademase-transitioning])**

4 had previously received pegademase while participating in the Phase 3 clinical trial[1] (referred to as the STP group [registered as clinical trial STP-2279-002])

1. Dorsey, M.J. et al. *J Clin Immunol.* 43, 951–964 (2023).

What did researchers learn from this study?

Was elapegademase effective in newborns that had never received ERT?

Researchers measured the levels of toxic dAXP caused by ADA deficiency and the levels of replacement ADA coming from elapegademase treatment.

Most newborns (85.7%) had high ADA levels and low levels of toxic dAXP. One baby with dAXP at a toxic level had been in the study for only 1.6 months before undergoing bone marrow transplant.

Did elapegademase promote health in patients who switched from pegademase treatment?

By the end of the study, **most patients (75%) had high ADA levels and low levels of toxic dAXP, including all STP patients and two-thirds of PT patients.**

Did patients have side effects while taking elapegademase?

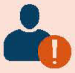

Like all medicines, elapegademase treatment can have side effects. The side effects that patients have while taking a medicine during a research study are known as adverse events (AEs).

Adverse events might be unrelated to treatment, or they may be related to the treatment being studied (elapegademase). These can be things like feeling sick, having an allergic reaction, or other health issues that occur during the research.

7 treatment-related AEs in 4 (12.5%) patients were deemed to be at least possibly related to elapegademase, and no treatment-related AEs were seen in the STP patients, who had been on elapegademase the longest.

In the EN group, 2 patients had thrombocytosis, 1 patient had neutropenia, and 1 patient had hypercalcemia. In the PT group, 1 patient had injection site erythema, exposure via skin contact and weight gain.

Adverse events are considered serious when they are life-threatening, cause lasting problems, or need hospital care. About half of patients had serious adverse events in this study, but none were related to elapegademase therapy. No patients had to discontinue treatment with elapegademase due to adverse events.

Did patients have many infections, or need to be hospitalized, during the research study?

Infections and hospitalizations are key outcomes because they show how well the immune system is working. If patients with ADA-SCID get a lot of infections or need to go to the hospital often, it means their immune system isn't protecting them properly.

Tracking these issues helps to understand how effective a treatment is at keeping patients healthy and preventing serious health problems.

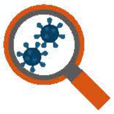

Treatment-emergent infections **occurred in 71.9% of patients**

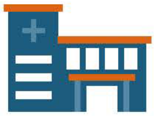

37.5% of patients had serious infections and required hospitalization

The **most common infection was COVID-19**, which emerged around the time the study began in late 2019 and was declared a pandemic shortly thereafter.

Did patients increase lymphocyte levels while being treated with elapegademase?

Researchers measured levels of lymphocytes in each patients' blood, and determined if those levels were improving over the course of the study. Stable lymphocyte levels indicated stable disease, or no progression or improvement.

In this research study, data were collected according to health care providers' routine clinical practice, and therefore lymphocyte levels were not always tested. Not enough data were collected to make a conclusion; however, patients in the **EN group had higher lymphocyte levels than other groups**, and patients who previously received pegademase appeared to have stable lymphocyte levels while taking elapegademase.

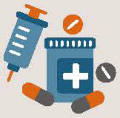

What did the study tell us and why is it important?

Elapegademase is a well-tolerated medicine for patients with ADA-SCID, especially for newborns and children. It helps maintain immune function and reduces the risk of severe infections. Despite the challenges of the COVID-19 pandemic, the drug was well-tolerated with no new safety concerns. This study supports the continued use of elapegademase for ADA-SCID patients who have taken pegademase in the past, and in newborns and children who have never been treated with ERT.

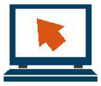

More Information

This summary is based on the article called "Multi-Year Registry Study of Elapegademase Treatment in Patients with Adenosine Deaminase Severe Combined Immunodeficiency (ADA-SCID) Requiring Enzyme Replacement Therapy", which was published in the *Journal of Clinical Immunology* in 2024. You can read more about the elapegademase real world study at https://classic.clinicaltrials.gov/ct2/show/NCT03878069.

This real world elapegademase registry study was previously sponsored by Leadiant Biosciences, Inc. and is now sponsored by Chiesi USA, Inc. Medical writing support for this summary was provided by Jackie L. Johnson, PhD of JLJ Consultancy B.V., which was funded by the sponsor in accordance with Good Publication Practice guidelines. The authors thank the patients and families who have contributed to the advancement of the understanding of ADA deficiency. Chiesi thanks Barb Ballard, SCID Angels for Life Foundation, for her editorial review of this summary. The original authors of the full article reviewed and approved this summary.
